# Human–Wildlife Interaction—A Social Survey

**DOI:** 10.3390/ani14050808

**Published:** 2024-03-05

**Authors:** Lara-Luisa Grundei, Franziska M. Schöttes, Friederike Gethöffer, Daniel Tost, Laurin Kluge, Ursula Siebert, Michael Pees

**Affiliations:** 1 Department of Small Mammal, Reptile and Avian Medicine and Surgery, University of Veterinary Medicine Hannover, Foundation, Bünteweg 9, 30559 Hanover, Germany; 2 Institute for Terrestrial and Aquatic Wildlife Research, University of Veterinary Medicine Hannover, Foundation, Bischofsholer Damm 15, 30173 Hannover, Germany

**Keywords:** human–wildlife interaction, urban wildlife, wildlife rehabilitation, wildlife care

## Abstract

**Simple Summary:**

The dedication of people towards wildlife will form an important part in future wildlife conservation and the halt of biodiversity decline. In this context, education and knowledge transfer can help to prevent actions deriving from misinformation. In this study, we refer to a survey among people interested in wildlife and try to highlight the importance of the improvement of educational services for a success in future coexistence and meaningful wildlife care. We discovered that a strong opinion about dealing with wildlife is associated with increasing contextual knowledge and experience in wildlife conservation.

**Abstract:**

With the results of a survey presented in this paper, we provide insight into public attitudes towards dealing with wildlife. Based on 1569 data sets derived from participating stakeholders, we inquired about the individual experience the participants had made with wild animals, and asked about their personal engagement, attitude towards management, and emotions involved and tried to evaluate basic contextual knowledge. As a result, we discovered a positive effect showing that a strong opinion about dealing with wildlife is associated with increasing contextual knowledge. People that are experienced in and engaged in wildlife conservation expressed significantly stronger positive emotions in this context. We conclude that education is essential in dealing with wildlife responsibly and that positive emotions are a main trigger for such engagement. The results of the survey underline that a combination of contextual knowledge and a positive attitude towards wildlife leads to a higher awareness of possible conflicts between humans and wildlife. Furthermore, these criteria are crucial when developing strategies that strive for a sustainable coexistence.

## 1. Introduction

Our anthropocentric-shaped century is causing a dramatically increasing loss of biodiversity, with 42,108 currently threatened species worldwide [1]. The main reasons for the biodiversity loss are changes in (agricultural) land use (e.g., habitat loss), urbanization, active exploitation like hunting and fishing, tourism, road constructions, climate change, environmental pollution, and invasive animal species [2,3,4]. In Germany, a densely inhabited industrialized country, urbanization leads to continuously expanding cities [5]. This trend can be observed worldwide, as estimates predict that one-third of the world’s population will live in urban regions by 2050 [6]. The progress of urbanization, agricultural land use, and the increasing infrastructure implicate that animals showing better adaptation strategies for living in close proximity to humans (synanthropic species) have discovered cities as a new environment, while those that have not (exoanthropic species) are being continuously driven out of their shrinking habitats [7,8].

Despite coping with human cohabitation whenever possible, synanthropic species are exposed to several negative environmental changes, including warmer climate, habitat fragmentation and loss, food depletion, disturbance, and pollution [9]. In cities as well as in rural areas, various factors can cause harmful effects on wildlife health. Depending on the environment, exposure to toxic substances can have an influence, but so can “new” predators as domestic cats and dogs, introduced diseases, and altered body condition. Further, architectural components in cities, for example, glass facades, are a huge mortality risk for avian wildlife [10,11]. A loss of approximately 100–115 million birds in Germany per year corresponds to 5–10% of all bird deaths occurring year-round in this country [12]. “New” predators kept as domestic pets like cats and dogs represent an additional threat for numerous species [13,14]. Roads are a key mortality factor for amphibian species, whose populations are steadily declining as a result of this [15], but also for other native wildlife [16,17]. Continuous structures like roads and railways prevent wildlife from crossing, causing habitat fragmentation [18]. Division of habitats and isolation of populations have serious negative impacts for population structure and health [19], as well as genetic consequences [20]. In contrast, urban green spaces may offer a chain of habitats in which many different animal and plant species live together and spread [21]. This partial heterogeneity can act as a driver for a diversity of species and enlarge the opportunity for animals to forage and find shelter [22,23]. Their ability to adjust and learn new behavioral strategies facilitates survival in a rapidly changing environment, possibly leading to a different activity pattern than their peri-urban and wild conspecifics [22,24,25,26]. Whether there is an increase or decrease in species diversity in urban regions reflects geographic and species-dependent variables; thus, a change in species composition along the rural–urban gradient is discernible [3]. In addition, for some species, it is confirmed that the population density increases and the home range size decreases in urban areas [27,28].

Due to the high population densities of some animal species and humans as well as their close proximity, interactions between wildlife and humans occur. These interactions are manifold and can lead to consequences for both parties, not always of an intentional nature. Positive benefits for humans when interacting with wildlife include recreational, educational, or psychological benefits [29]. Negative interactions, so-called human–wildlife conflicts, can occur as emotional or economic conflicts as well as risks for human health [30]. Interaction with injured wildlife sometimes can be misinterpreted and could therefore harm the animals more than help them. Every spring, young wild animals, most often fledging birds, are brought to veterinarians or wildlife sanctuaries because people are convinced they need help [31]. Furthermore, rescuers sometimes try to rear wild animals like chicks at home with little knowledge about their appropriate needs [32]. In many countries, like Germany, it is therefore prohibited by law to take healthy wild animals from the wild and it is mandatory to have the necessary knowledge and skills regarding appropriate nutrition, care, and housing when care is needed [33,34].

Measures are needed to avoid conflicts and negative consequences and to guide the interaction with wildlife in a rational way. There are diverse opinions and interests represented in the population at large about the necessary measures to be taken [35]. Interests range from animal welfare, species protection, and environmental protection to economic interests, traditional animal disease control methods, management, human health protection, and administrative concerns [36,37,38]. In the context of the modern attitude towards animal protection and wildlife conservation, it is essential to discuss how society wants to deal with wildlife ethically, emotionally, and practically in a changing world and relating to the aspects of climate change and biodiversity loss. Finding answers to these questions is important for the future development of urban planning and even more so for the future development of society and wildlife.

A social debate funded by the federal state of Lower Saxony, Germany, was launched to identify the interest groups and compile initial impulses of all participants on how to deal with native wildlife in urban surroundings in future. This included a survey to explore social opinions of all interest groups involved in this topic and to establish a basis for recommended approaches in wildlife management. Therefore, our research aimed to present the results of this survey in the context of the human–wildlife conflict debate. For further evaluation, we established three hypotheses:

First, we hypothesized that the opinion towards wildlife management and handling was influenced by personal factors, contextual knowledge about wildlife, own experience with or engagement for wildlife, and associated emotions.

Second, we assumed that emotions associated with wildlife were influenced by personal factors and own experience with and engagement for wildlife.

In the third hypothesis, with respect to the occurrence of wildlife in the surroundings of participants, we presumed that diversity indices of wildlife species, as well as the living place according to residential density, had influence on defined scores regarding emotion, engagement, experience, opinion, and knowledge.

## 2. Materials and Methods

### 2.1. The Study Setup

We designed an online questionnaire in German containing 40 questions using the software LimeSurvey (LimeSurvey GmbH, version 3.23.1+200825, Hamburg, Germany). We defined wildlife as “free-ranging vertebrates in Germany” including feral pigeons and excluding strays. Feral pigeons are not officially defined as “wildlife” as they are former feral domestic breeds [39,40], but we decided to include them because they belong to the people’s perception of wild animals, especially in an urban environment.

The main study region included the whole of Germany. The survey was publicized among the general public, intending to address stakeholders and the part of society interested in wildlife. These included people who came into contact with wildlife on a regular basis, either professionally or voluntarily, e.g., veterinarians, wildlife sanctuary keepers, wildlife volunteers, animal welfare and species conservation associations, veterinary offices, and municipal agencies as well as professional or amateur hunters. The focus of the survey should not be on a representative cross-section of the general public but on the attitudes of the various stakeholders who were already involved with wildlife.

Subdivided into six sections with an optional seventh block for wildlife volunteers, the first section of the survey asked questions about personal data like gender, age, profession, hunting license or volunteer work, and a ZIP code of the hometown as a reference for its population density. The second section tested the knowledge of participants about legal issues, responsibilities, and wildlife diseases using “quiz”-type questions. In the third section, the questions aimed to outline the experiences the participants had had with wildlife, both positively and negatively. Next, we asked about people’s feelings towards specific animal species and their engagement opportunities. The fifth section focused on the opinions of the participants towards various issues and measures regarding wildlife management. Subsequently, we asked about own past and future engagement regarding wildlife. The optional seventh section was a detailed survey for wildlife volunteers. Here, we asked for details of their work and the main challenges they faced. All questions and answer options are presented in Table S1.

We used single and multiple-choice structures, depending on the aim of the question and relevant answer options. Some questions could be answered with yes/unsure/no; for some answers, we asked the participants to rate their responses according to a scoring system. This could be rating between one (very negative) and five (very positive). Other questions required different types of scales with options from strongly agree/agree/partly agree/partly disagree/disagree/strongly disagree or applies completely/applies/partly applies/does not apply/does not apply at all [41]. Some questions could be answered by choosing a predetermined response. We also used follow-up-questions when a specific answer was given. Every question could either be skipped or contained the option “no answer”. The questionnaire was available online for 10 weeks (4 August 2022–12 October 2022).

### 2.2. Data Preparation and Evaluation

All questionnaires were evaluated descriptively using Microsoft Excel 2016 (Microsoft Corporation, Redmond, WA, USA). Answer options with gradations such as “agree completely” to “disagree completely” or ranking questions were recoded to an ordinal system (e.g., 1–5) to display the median results. We assigned personal data like year of birth or ZIP code to different groups for the evaluation, e.g., age groups and residence according to population size of hometown (see Table S1).

For statistical implementation of the hypotheses, we summarized answers on similar thematic items as total scores [36,42]. Therefore, we recoded data according to their thematic approach leading to the following scores (for details, see Table A1):

1. Emotion score (EmS): This included two questions asking the participants about their feelings towards species and about negative emotions like fear or disgust regarding wildlife.
2. Engagement score (EnS): This consisted of three questions related to the participants’ past and future commitment to wildlife such as financial, active or political involvement or how much financial support would be granted for the care of a wild animal in need.
3. Experience score (ExS): This included three questions about the types of interactions with wildlife that had been experienced, whether any wildlife had ever been found in distress, and, if so, what action had been taken.
4. Knowledge score (KS): This consisted of four questions from the “quiz” section, containing questions about the responsibilities, legal framework regarding wildlife, and zoonotic diseases.
5. Opinion score (OS): This included two questions asking for agreement or disagreement with various exemplary ways of dealing with wildlife, e.g., handling referred to reintroduction, euthanasia for animal welfare reasons, and the keeping of wild animals.

Recoding for statistical evaluation was performed either from +2 to −2 (if five options to answer were available, e.g., strongly agree to strongly disagree), +1 to −1 (for yes/unsure/no) or +1 to zero (for right or wrong answers). For questions that asked about experiences or feelings, negative feelings were assigned according to negative numbers and positive feelings according to positive numbers.

Information on presence of wildlife species was obtained from the Wildlife Monitoring Survey for Lower Saxony (WTE; “Wildtiererfassung”) provided by the Federal Hunting Association of Lower Saxony (LJN). This dataset contains occurrence data of various game species given in percent per administrative unit (ZIP code). Diversity indices were calculated using the Shannon index (H’) for all species surveyed (see Table S3) and separately for grouped species (mammals, predators, ungulates, ducks and geese, feathered game, and neozoa). As wildlife data were only available for Lower Saxony, only responses from participants in that federal state (n = 279) were considered in this analysis. To check for the quality of the scores, which consisted of several items each (see Table S2), we used the statistical index of Cronbach’s α [43]. Cronbach’s α describes the internal consistency of a scale, indicating how well the summarized items are appropriate to the respective score [44]. The quality of the score is considered acceptable with a Cronbach’s α value of 0.7 or more.

### 2.3. Statistical Analysis

For statistical modeling, we fitted Generalized Linear Models (GLM) with Gaussian error structure and identity link function and performed Analysis of Variances (ANOVA) on fitted GLMs. The OS as well as the EmS functioned as dependent variables for hypotheses one and two, while demographic variables and all other scores were used as independent variables with interactions. For the third hypothesis, we used the scores as dependent variables and Shannon index (continuous) as well as residence (categorical) as independent variables with interaction. We tested the variables for correlation and based the model selection on a good explanation of the effects, the Akaike Information Criterion (AIC) [45], and one, if possible, simple but meaningful model design. The model checks were also carried out using QQ plots of the residuals, the residual distribution, and the leverage effect of outliers [46]. For hypothesis three, we linked the score results to diversity indices of wildlife species and population size of hometown via participants’ ZIP codes.

We used R studio 2.1 and R 4.1.0 (https://posit.co/products/open-source/rstudio/ accessed on 6 January 2023, [47]) for all descriptive and statistical calculations and specified the level of significance as 0.05.

## 3. Results

### 3.1. Participants

**Figure 1:**
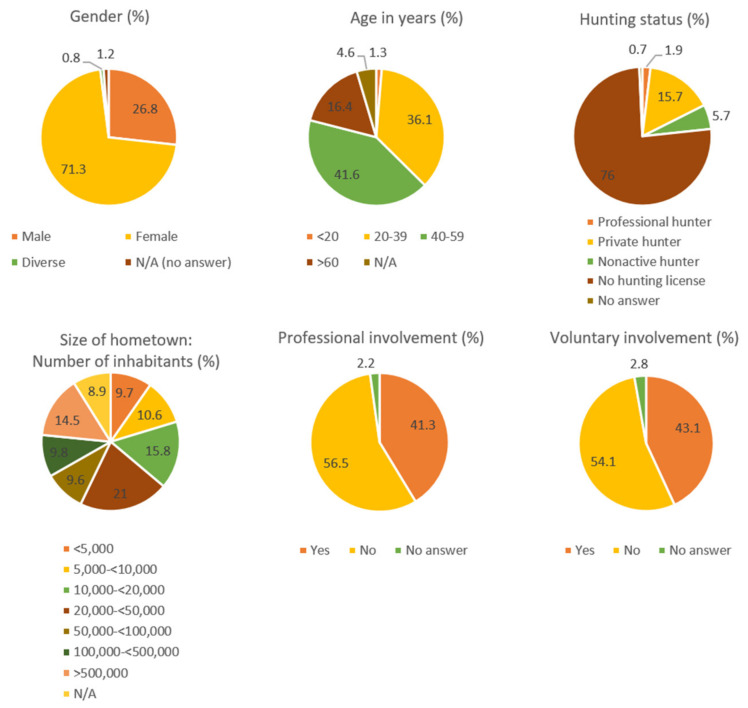
Descriptive analysis of the participants’ (n = 1569) background, including personal data concerning gender, age, and size of hometown, as well as information on wildlife-related activities such as hunting license and voluntary/professional involvement (relative proportions).

In total, 2067 people participated in the survey. The database was cleaned up to 1569 complete contributions (see Figure 1 for detailed information about the participants). The median year of birth of contributors was 1977, with the youngest born in 2006 and the oldest in 1938. A very small number of participants (n = 29) in the survey indicated that they did not live in Germany. Of those who dealt professionally with wildlife, the majority worked in veterinary medicine or animal care (n = 361), while the minority worked in agriculture (n = 18). The participants who were voluntarily involved with wildlife received an additional block of questions about their volunteer work at the end of the survey.

### 3.2. Emotion

**Figure 2:**
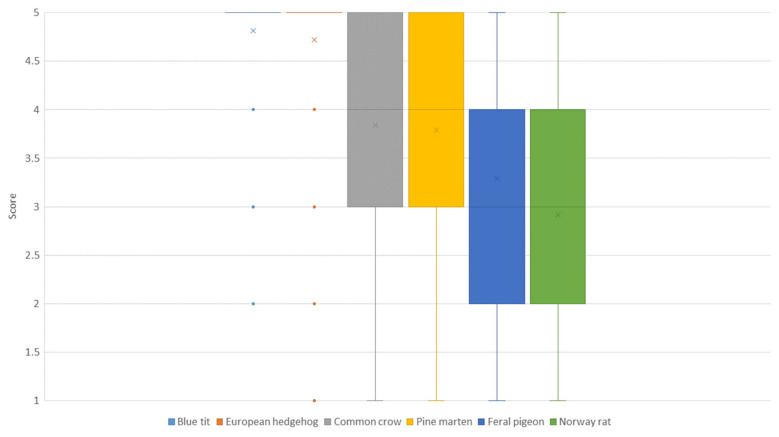
Results for the question about emotions towards wildlife: Rate the following animal species according to your feelings towards them (Matrix: very negative (1)/rather negative (2)/partly positive, partly negative (3)/rather positive (4)/very positive (5), n = 1569). Median and mean indicated with a line (─ and a cross (x), respectively. The figure contains 6 out of 10 asked for animal species: blue tit (*Cyanistes caeruleus*), European hedgehog (*Erinaceus europaeus*), common crow (*Corvus corone*), pine marten (*Martes foina*), feral pigeon (*Columba livia* forma domestica), and Norway rat (*Rattus norvegicus*).

**Figure 3:**
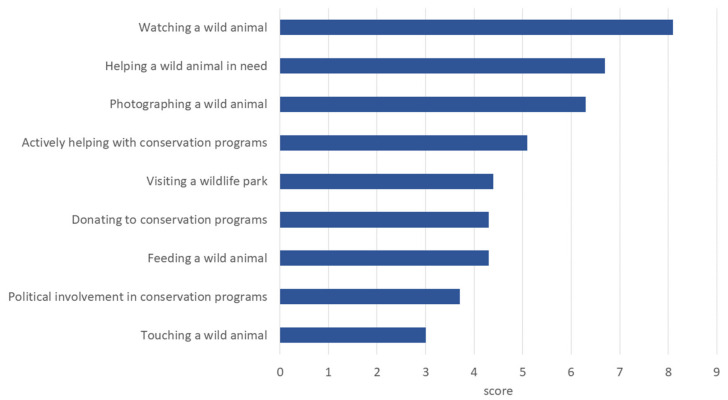
Results for the question about value of interactions: Sort the following interactions in descending order according to how you value them and the feeling of happiness associated with them. (Rank 1 to 9, n = 1569).

Overall, emotions towards wildlife were mostly positive (median emotion score 19, range −28 to +28, Table 1). When asked about their emotions towards different species, the participants showed diverse feelings about them (see Figure 2). As shown in Figure 3, experiences with individual interactions (range 1 to 9, Table S1), like watching (average 8.1) or helping (average 6.7), brought the respondents a greater feeling of happiness on average than being involved in a more indirect way like donating (average 4.3) or political involvement (average 3.7). Most of the participants did not experience any strong negative feelings (range 1 to 5, Table S1) towards wild animals such as fear (e.g., fear of attack: median value 2) or disgust (median value 1).

### 3.3. Engagement

**Figure 4:**
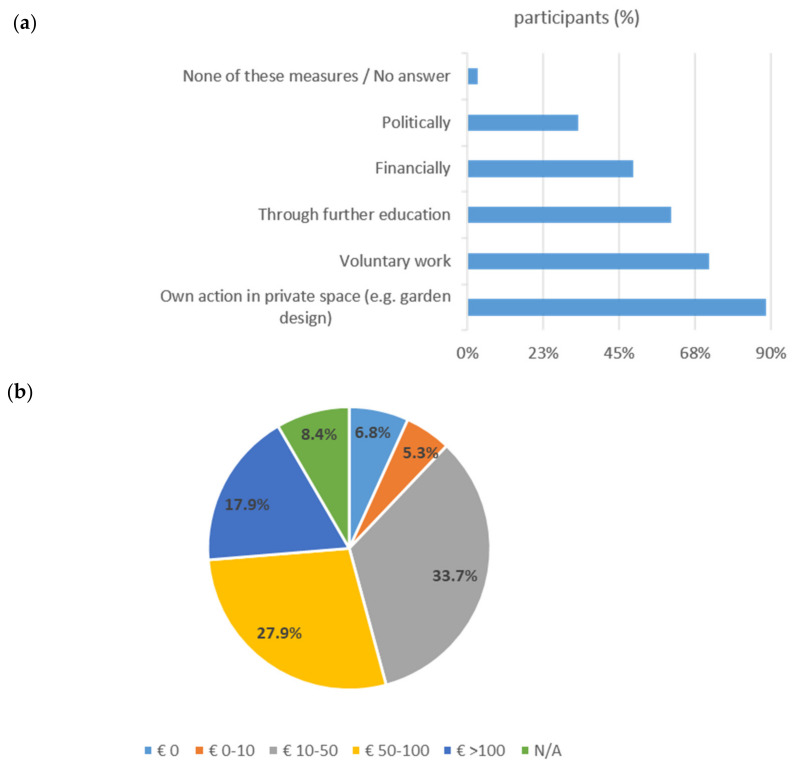
Results for the questions about engagement (n = 1569): (**a**) To what extent would you be willing to get involved with native wildlife in the future? Percentage of agreement shown on x-axis. (**b**) How much money would you be willing to pay to a veterinary practice/wildlife center for the care of a wild animal you have rescued? Percentage of financial amount shown in the graph.

Most of the respondents had been involved in native species conservation programs, whether through donations, active help, monitoring, or political commitment (median engagement score 6, range −1 to +10, Table 1). The willingness to become involved in the future was related more to private initiative and less to political activism (see Figure 4). When asked about their willingness to pay for (medical) care of a wild animal, more than half (61.6%) of the participants were willing to pay between €10 and €100 for the care/treatment, as presented in Figure 4.

### 3.4. Experience

**Figure 5:**
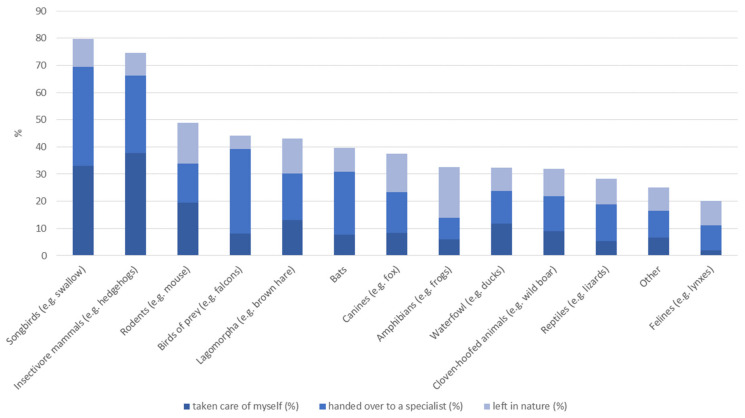
Results for the question about found wildlife species: You stated that you found a wild animal in need of help once or several times. Please indicate how you reacted for all applicable animal groups. (Matrix, follow-up question if C3 was answered with “yes”, n = 1146).

The median experience score was 5 (range −4 to +17, Table 1), with negative experiences recoded negatively and positive experiences recoded with positive numbers. More than 99% of the participants had had encounters with wildlife, of which most were positively assigned such as observation (97.7%), feeding (63.9%), and touching (65%). Fewer encounters were negatively associated like damage (27.3%), zoonosis (6.6%), attack (6.2%), or harassment (7.8%) by wild animals. Nearly three quarters (73%) of the respondents had also found a wild animal in need of help, mostly hedgehogs (74.6%) and songbirds (79.7%); see Figure 5 for detailed reactions to the aforementioned situations differentiated according to animal groups.

### 3.5. Knowledge

**Figure 6:**
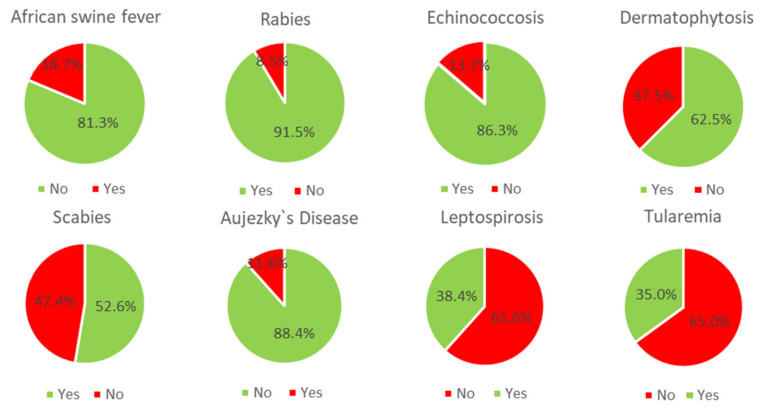
Results for the question about zoonotic diseases: Which of the following diseases belong to zoonoses? (Zoonoses = diseases that can be transmitted between animals and humans), green = right answer, red = wrong answer, n = 1569.

Questions about responsibilities for wildlife, legal framework, and zoonotic diseases (see Figure 6) were mostly answered correctly (median knowledge score 10, range 0 to 14, Table S2). Of 1569 participants, 39.4% knew that the person who rescues an injured wild animal that is not subject to hunting laws is responsible for this animal. Over one third (31.6%) of respondents selected the wrong answer: “veterinary office”.

### 3.6. Opinion

**Figure 7:**
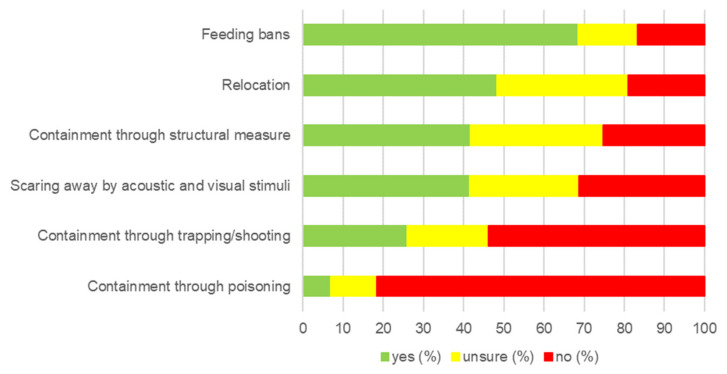
Results for the question about management measures: If necessary, do you consider the following measures to be justified to protect against wildlife damage in urban areas? (Matrix, n = 1569). Percentage of agreement, abeyence, or disagreement shown on x-axis.

Opinion score (OS) ranged from −14 to +14, expressing maximal agreement with handling of wildlife, including management tools with a high score, and disagreement with handling by humans with low scores. Among all responses, a moderate handling was expressed most frequently, resulting in a median score of 3 (Table 1). The participants mostly agreed with statements about rehabilitation of wild animals (range 1 to 5, Table S1) for animal welfare reasons (median value 5) and permanent keeping of wild animals for species conservation purposes (median value 4) but were unsure about euthanasia of an animal that cannot be released back into the wild (median value 3). Most problems in the interactions between wildlife and humans (range 1 to 5, Table S1) were seen in the lack of knowledge or lack of education concerning wildlife (median value 5) and habitat overlap (median value 5). When it came to having to take measures in the context of wildlife management, people preferred the indirect and less invasive strategies, as shown in Figure 7.

### 3.7. Volunteer Questionnaire

**Figure 8:**
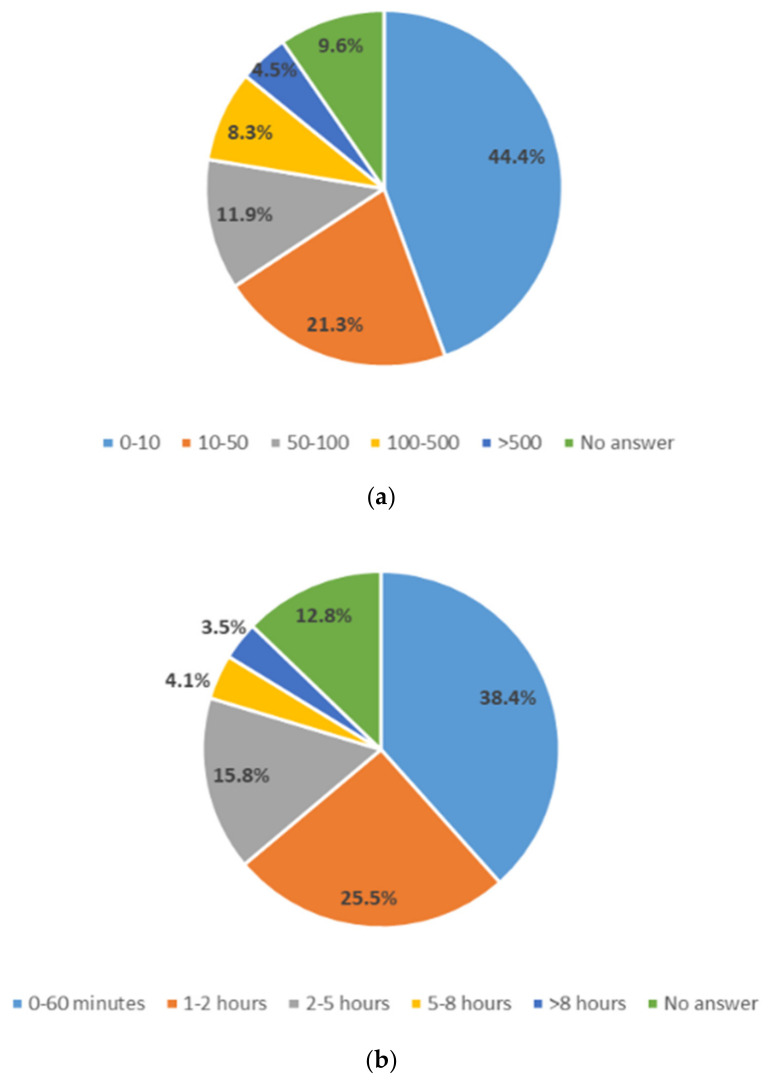
Volunteers in wildlife practice: (**a**) estimated wildlife cases per year (n = 666), (**b**) estimated time spent per day per animal (n = 664).

This optional section was completed by 671 participants who were volunteering in wildlife care programs (see A6 in Table S1). According to this survey, songbirds (Passeriformes, n = 344), insectivores (Eulipotyphla, n = 312), and rodents (Rodentia, n = 173) were among the three most common animal groups of wild animals taken in and cared for. Most volunteers financed costs through private funds (82.6%). Of the volunteers surveyed, 81.7% stated that they would welcome mandatory accredited further training for volunteer wildlife carers. The estimated number of individual animals taken in per year and the time spent caring for each animal per day are displayed in Figure 8.

**Figure 9:**
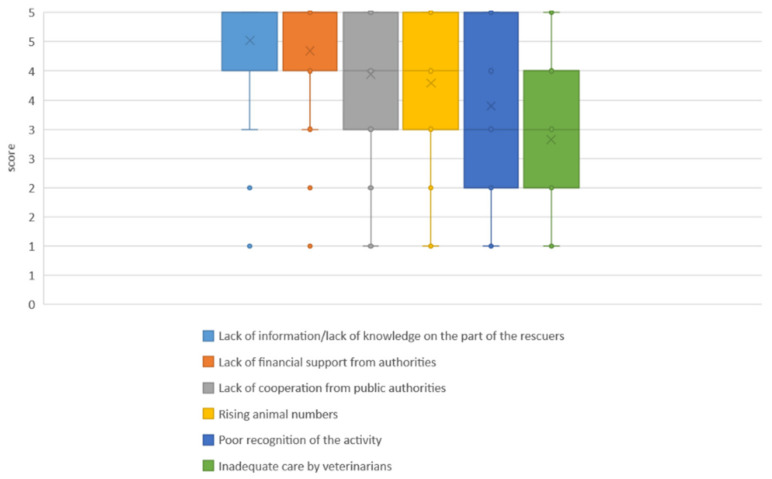
Results for the question about issues affecting the work of volunteers (Matrix: this does not affect my voluntary work at all (1)/this does not really affect my voluntary work (2)/this partly affects my voluntary work (3)/this affects my voluntary work (4)/this greatly affects my voluntary work (5), n = 644). Median and mean indicated with a line (─) and a cross (x), respectively.

**Figure 10:**
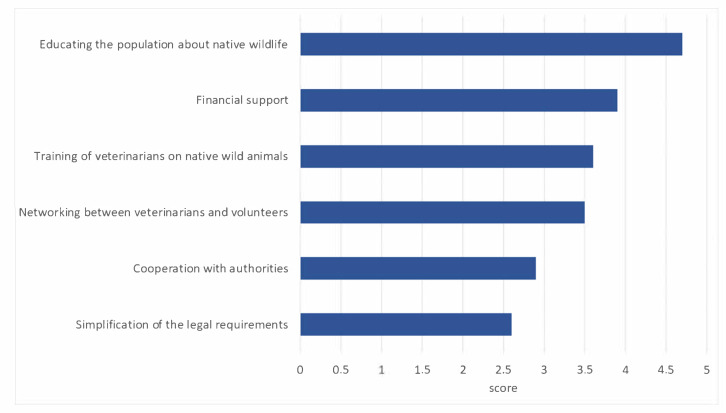
Results for the question about improvement request: What do you think should be improved most urgently in the future? Sort the sentences in descending order of importance. (Ranking 1–6, n = 671).

When asked about the largest problems affecting their work, the respondents chose the option the lack of information or lack of knowledge on the part of the animal rescuers the most (see Figure 9). This was also reflected in the question about the greatest desire for change (Figure 10).

### 3.8. Statistical Score Evaluation

The opinion score (OS) as well as the engagement score (EnS) reached an acceptable Cronbach alpha value (see Table 1). For emotion score (EmS) and experience score (ExS), a good Cronbach’s α could be achieved. Although the knowledge score only received a poor outcome in Cronbach’s α, we nevertheless decided to keep it in the model due to its contextual importance.

**Figure 11:**
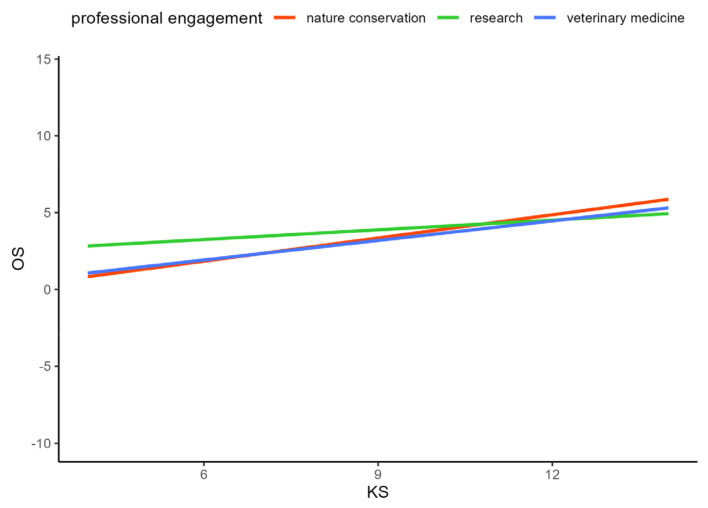
Differentiated professional engagement of the participants in interaction with the opinion score (OS) and knowledge score (KS).

**Figure 12:**
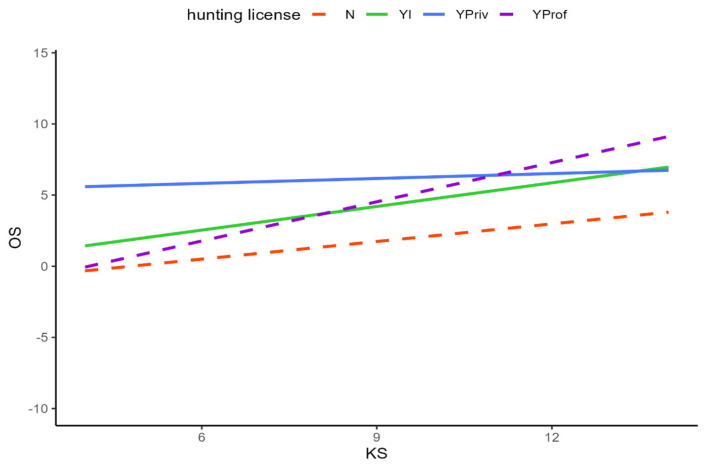
Differentiated hunting status of the participants in interaction with the opinion score (OS) and the knowledge score (KS). N = no hunting license, YI = nonactive hunter, YPriv = private hunter, YProf = professional hunter; dashed lines represent participants that are active in hunting.

When relying on the best model and also respecting interactions between some of the factors, the opinion score (OS) was significantly higher if people had also attained a high knowledge score (KS, *p* < 0.0001), were working professionally with wildlife in the field of veterinary medicine, nature protection, or research (*p* < 0.0001, Table A2, Figure 11), or held a hunting license (*p* < 0.0001, Table A2, Figure 12). Higher concerns about damage to and diseases derived from wildlife, or the assumption that society discussed wildlife topics too emotionally, led to a higher OS again. On the contrary, significantly lower OS could be seen among female respondents (*p* < 0.0001, Table A2), persons that did not work (voluntarily) with wildlife (*p* < 0.024), those that also expressed a high emotion score (EmS) in the survey (*p* < 0.0220), or persons that stated to have stronger concerns about missing knowledge or prejudice about wildlife among the public (*p* < 0.0004, 0.0014, respectively). All results are shown in Table A2.

**Figure 13:**
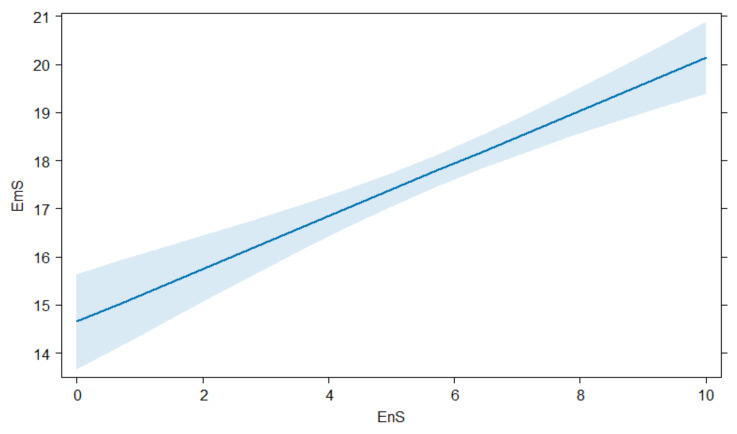
Linear regression showing an increase of the emotion score (EmS) and the engagement score (EnS) among survey participants.

Regarding EmS, we could demonstrate that people with a high experience score (ExS) or engagement score (EnS, see Figure 13), people that had no hunting license or were not active hunters as well as those living in towns with fewer than 50,000 inhabitants reached significantly higher EmS, while the knowledge we asked about in this study (KS) had no significant influence on these results. Male participants as well as persons working in the agricultural field achieved significantly lower EmS. All results are shown in Table A3.

The knowledge score (KS) correlated with the calculated Shannon index of game species surveyed in the WTE, but not with the living place according to residential density. With increasing wildlife diversity (H’), the KS also increased significantly (*p* = 0.02), but at a low level. This effect was especially true for occurrences of feathered game (groups: geese and ducks (*p* = 0.0006); neozoa (*p* = 0.02); birds (*p* = 0.0008); see Table S3). On the other hand, diversity of mammals and groups dominated by mammals (ungulates, predators, management species) showed no significant effect on the KS. All other scores (EmS, EnS, and ExS) did not correlate with calculated diversity indices or residential density.

## 4. Discussion

Social research can be conducted using a variety of methods, particularly the survey format [48]. Including our survey in the scope of an externally funded project enabled us to reach out to stakeholders as well as to the interested general public. Using digital and remote tools, we managed to reach a large number of participants, mainly located in Germany. The study effectively represents all of the main interest groups, especially those who are involved in wildlife health and management. Our main objective was to represent the intersection of all who relate to wildlife in a private, professional, or interest-related way, and not to obtain a cross-section of the total German population. The results therefore express the genuine opinion of dedicated people working in the field of wildlife rehabilitation, where different interests can lead to a challenging social debate.

Our hypotheses were partially confirmed and partially refuted, as described in more detail below.

*Knowledge as a key role*

In this survey, we found opinions towards wildlife handling and management (OS) to be particularly correlated with contextual knowledge (KS) and a professional (veterinary medicine, nature conservation) or private (hunting license) interest in wildlife, as hypothesized previously. When interpreting the scores, it is important to note, for example, that a high OS does not mean that the opinion on wildlife is very positive, but that there is a stronger agreement on handling and management measures regarding wildlife. This illustrates that a rational background promotes consent to handling of wildlife and vice versa [49]. Concerns about lacking knowledge about wildlife are related to lower approval of management measures. This may result from a reflective perspective on human–wildlife interactions and their consequences, leading to a more restrictive attitude towards (invasive) management measures. The same is true for those respondents holding concerns about prejudice against wildlife. Negative attitudes towards and prejudices against wildlife seem to be studied far more frequently in research than positive attitudes. Antipathy attitudes towards wildlife may be justified due to cultural and religious rites, traditional negative stimuli (e.g., spiders) [50], spiritual beliefs [51], or genetically based phobias [52], among other things. In the literature, it is also known that some groups of animals have lower rankings in public evaluation, such as invertebrates. This may be due to a fear or disgust of the species, for reasons of infectiousness, aversion to mucus, or other characteristics, such as the inability to identify these species [53,54]. As shown in our data, there were also vertebrate species, like brown rats and feral pigeons, that still have a bad reputation. This can lead to a preference for aversion of welfare-friendly measures by the general public, for example, in feral pigeon management [55]. In contrast, a more positive view of people towards larger vertebrates, especially mammals and birds, is reported [56].

In this context, those participants actively involved with anthropomorphism of human–wildlife contact have a stronger opinion towards handling and management of wildlife. A more rational than emotional way of thinking, as well as interpreting a need to prevent negative effects caused by wildlife, could be the reason for this. It becomes clear that contextual knowledge plays a key role in forming a substantiated opinion. Since a correlation between higher levels of education and a positive attitude and esteem towards wildlife is described [57], it can be assumed that education about wildlife can reduce prejudices against and anthropomorphism of certain species. In a democratic society, pluralism is a cornerstone, but the main sources of information for forming opinions should be based on scientific facts and rational thinking. The lack of education and the overlap of habitats are known to be a major problem in human dimensions research [58].

The opportunity to interpret the survey in the light of biodiversity indices had to be taken. Nevertheless, most probably due to the relatively small number of participants regarding different regional origin, this evaluation did not lead to significant outcomes, thus refuting our third hypothesis. However, we observed the trend that better-informed participants (higher KS) lived in regions with a slightly higher biodiversity indices. It will be necessary to enlighten species richness and presence in future research.

*Emotions as a driver for engagement*

The willingness to commit to nature and species conservation through own actions has declined in recent years [59]. Our hypothesis that the willingness to engage with wildlife is related to positive feelings towards them was confirmed. This was supported by the fact that action in connection with an individual animal (helping, observing) was attributed to a higher value than in combination with species conservation projects. Although a high number of individuals are involved in these projects, they merge anonymously into an indistinguishable mass (C5, Table S1). Such a strong attachment to individual animals can be found in the literature as well [56]. This is also similar to findings that describe that the willingness to implement small behavioral changes is higher than actively being involved in a nature conservation association or donating to environmental protection causes [59,60].

When considering the gender of the survey participants, females made up the largest proportion, especially in the field of veterinarians and volunteers. Their involvement in individual animal fates could lead to a lower support of general management measures (OS). This becomes particularly obvious since these participants also reached a high EmS. However, it must be noted that extremely positive feelings towards animals like songbirds (blue tit) and hedgehogs can lead to humanized actions, for example, animals being rescued even though not in need of help or being fed with (in)appropriate food [61]. Songbirds and hedgehogs are associated in the public with purely positive emotions; they can be observed in the garden without posing a danger, and they are the animals that are most often rescued (songbirds n = 913; hedgehogs n = 855). Often, very strong affection and concern can be seen in the general public, reaching romanticized ideas in relation to animals and nature [56]. It is known that such feelings depend on factors such as beauty, usefulness, and rarity of a particular species [51]. External influences and personal behavior patterns are also known to be a determining factor in our favorability or unfavourability towards species and can be understood as instrumental factors [56]. Therefore, it is important to raise awareness of the needs and requirements of wild species in order to prevent inappropriate handling.

Interestingly, respondents without a hunting license or without active hunting practice showed a higher EmS. This could be explained by the lack of experience and a different understanding of flora and fauna, which practicing hunters might possess. When looking at the aspect of occupational background in connection with the emotion score (EmS), we noticed that people who work in agriculture had the lowest EmS. It is obvious that when animals are kept for one’s own livelihood and products are obtained from them, a rational rather than emotional perspective is taken, since the animal is not treated as a companion animal or family member but is used economically [62]. In philosophy, this phenomenon is called speciesism and means “a prejudice or attitude of bias in favor of the interests of members of one’s own species and against those of members of other species“ [63].

The median experience score was low (median value 5, range −4 to +17, Table 1), although more than 99% of respondents had had experience (positive or negative) with wildlife in the past. Although negative experiences were coded with negative numbers and positive ones with positive numbers, respondents had not had predominantly negative experiences with wildlife, but rather their experiences had been limited to a small selection of the proposed wildlife species. Furthermore, the respondents tended to leave certain species in the wild rather than intervene, like reptiles, which were either cared for in private or taken to a specialist a total of 215 times only. In contrast, an insectivorous mammal like the hedgehog was taken care of 759 times in our survey.

In summary, it is important to recognize that engagement for and experience of wildlife are inevitably related to emotions towards it. We have to acknowledge that individually experienced interactions with an animal may have a greater impact on personal emotions than a more abstract and distant management of animal populations that people are mainly informed about.

*How to deal with wildlife in the future?*

Based on the results of this survey, we anticipate that the high motivation of people to contribute to the protection of native wildlife through own actions and behavioral changes should be channeled into meaningful paths through education campaigns and political measures.

The more facts people are aware of, and the deeper the knowledge among professionals, the better prejudices can be diminished and humanization prevented [64]. Verbal exchanges between professionals and the general public enable a social discourse that contributes to the improvement and forming of public opinion. Political and private initiatives should develop recommendations for actions and cite positive examples to offer guidance. Suggestions and initiatives, for example, for wildlife-friendly garden designs and public relations, could prevent unnecessary rescuing of animals and would benefit native wildlife. Known pioneer concepts such as “wilderness in cities” [65] or the “animal-aided design” [66] describe approaches to urban design that consider wildlife and offer successful solutions. Following the survey and the project content, recommendations for action on how to deal with wildlife were drawn up from the scientific findings, which are intended to provide a guideline for the establishment of a nationwide wildlife management system for Germany [67]. The federal state of Baden-Württemberg is a pioneer in wildlife management and wildlife–human conflict solution with the appointing of “wildlife officers” in large cities [68]. Such and similar recommendations for action are needed to formulate common goals and integrate wildlife in our environment. Cooperation between the individual stakeholders, in which common goals are prioritized over conflicts of interest, is urgently needed [30]. Well-communicated strategies about management and handling can lead to a widely accepted approach to wildlife. Supportive rules of conversation [69] or mediation are recommended here. In the same way, publicly communicating knowledge is a prerequisite for further development.

Individual wildlife protection and wildlife conservation do not always coincide, but they complement each other in many ways. The positive emotions felt for wildlife are important for a commitment that is urgently needed in the field of animal and species protection. An overlapping collaboration of both disciplines is crucial for sustainable management of our native wildlife as well as knowledge being indispensable as a basis for professional handling of wild animals.

*Limiting factors*

When evaluating our data, it must be taken into account that the sample size reflects the general public with professional or private interest in wildlife, as a disproportionately large number of people from the veterinary medicine and animal care sector (n = 391) took part in the survey. An increased participation of people with a certain affinity to the topic is often observed in opinion studies [70]. As the study was only available in German, the study area was limited to German-speaking countries.

On the other hand, the KS was relatively weak, which could be caused by the complexity of the survey and variety of question types. This sometimes made it difficult to compare individual questions with each other but made it possible to ask the questions in more detail. Even if this can make it seem more monotonous for the respondents, a standardization of the question types would have contributed to a better score formation.

## 5. Conclusions

Wildlife research is experiencing rising interest with regard to human–wildlife interactions in Germany, with an increased research focus and number of publications [71,72]. Lessons learned when integrating the wider community can be used as a basis to develop strategies for the future coexistence of humans and wildlife.

In our study, it became clear that people’s behavior toward wildlife depends on their personal experiences, engagement, and feelings. Therefore, it is crucial to guarantee early wildlife education as a basis for an animal-friendly approach to wildlife. Education and experience enable people to act rationally and professionally, which can lead to a social change towards coexistence with wildlife, not only in urban areas. Communication campaigns must be considered in management endeavors, and educational work needs more attention among political decisions. Before political measures can be taken in favor of wildlife and species protection, the general public must be sensitized to this issue.

The results of this survey highlight the importance of emotions in combination with engagement and experience on the basis of knowledge and their role in opinion formation and willingness to become involved with native wildlife. Education and awareness are the key to a society capable of action. To secure the future coexistence of humans and wildlife, we would welcome a motivated implementation of our recommendations and a willingness to further develop a consensus on a societal and professional level.

## Figures and Tables

**Table 1:** Score calculation, minimum, maximum, median, and Cronbach’s alpha value.

Scores	Min.	Max.	Median	Raw Cronbach’s Alpha
Emotion (EmS)	−9	28	19	0.8
Engagement (EnS)	0	10	6	0.7
Experience (ExS)	−2	17	5	0.8
Knowledge (KS)	4	14	10	0.5
Opinion (OS)	−10	14	3	0.7

## Data Availability

The data presented in this study are available on request from the corresponding author.

## Supplementary Materials

The following supporting information can be downloaded at: https://www.mdpi.com/article/10.3390/ani14050808/s1, Table S1: Output of questionnaire used for this study; Table S2: Variables used in the statistical model; Table S3: Grouped wildlife species for wildlife survey dataset.

## Appendix A

**Table A1:** Information about scoring: Included questions and answer options. N/A is given when no answer was given.

Score	Question	Answer Options
*Emotion score (EmS)*	Rate the following animal species according to your feelings toward them (1–5).	Norway rat (*Rattus norvegicus*) Feral pigeon (*Columba livia* forma *domestica*) Pine marten (*Martes foina*) Blue tit (*Cyanistes caeruleus*) Red squirrel (*Scirurus vulgaris*) Common crow (*Corvus corone*) European hedgehog (*Erinaceus europaeus*) Common buzzard (*Buteo buteo*) Mallard (*Anas platyrhynchos*) Slow worm (*Anguis fragilis*)
	To what extent do the following feelings about some wildlife apply to you? Please rate (1–5).	Fear of illness/risk of infection Fear of attack Fear of harassment/harm Disgust
*Engagement score (EnS)*	Have you ever been privately involved in native species conservation programs?	Yes, financially Yes, voluntary work/active participation Yes, monitoring/observations Yes, politically No N/A
	To what extent would you be willing to get involved with native wildlife in the future?	Financially Voluntary work Politically Through further education Own action in private space (e.g., garden design) None of these measures N/A
	How much money would you be willing to pay to a veterinary practice/wildlife center for the care of a wild animal you have rescued?	€0 €0–10 €10–50 €50–100 €>100 N/A
*Experience score (ExS)*	What kind of encounters have you had with wildlife so far?	Observation Feeding Touch Damage Zoonosis Attack Harassment I have not had any encounter N/A
	Have you ever found a wild animal in need of help?	Yes No N/A
	You stated that you have found a wild animal in need of help once or several times. Please indicate how you reacted for all applicable animal groups. (taken care of by myself/handed over to a specialist/left in the wild)	Canines (e.g., foxes, raccoon dogs) Felines (e.g., lynxes, wildcats) Lagomorpha (e.g., brown hares, wild rabbits) Rodents (e.g., mice, dormice) Insectivore mammals (e.g., hedgehogs) Songbirds (e.g., swallows, crows) Birds of prey (e.g., falcons, owls) Waterfowl (e.g., ducks, geese) Bats Cloven-hoofed animals (e.g., roe deer, wild boar) Reptiles (e.g., lizards, turtles) Amphibians (e.g., frogs, toads) Other
*Knowledge score (KS)*	Who is legally responsible for an injured wild animal that is not subject to hunting laws?	The person who finds and takes the animal Veterinary Office Veterinarians Fire brigade Police Animal shelter N/A
	When is it legal to take a wild animal from the wild?	For permanent care when it would no longer cope in the wild For optimal, more pleasant wintering For health care and reintroduction into the wild If the animal is in danger from predators N/A
	Are veterinarians obliged to treat wild animals free of charge?	Yes In case of emergency No N/A
	Which of the following diseases belong to zoonoses? (Zoonoses = diseases that can be transmitted between animals and humans)	African Swine Fever Rabies Echinococcosis (Fox tapeworm) Dermatophytosis (skin fungus) Scabies (Mites) Aujeszky’s Disease Leptospirosis Tularemia (rabbit fever) N/A
*Opinion score (OS)*	To what extent do you agree with the following statements? (1–5)	The care and reintroduction of an injured wild animal makes sense for animal welfare reasons. An injured wild animal that cannot be reintroduced to the wild should be euthanized for welfare reasons. The keeping of native wild animals in wildlife parks is justified for educational purposes The keeping of native wild animals in specialist facilities is justified for species conservation purposes (breeding programs, reintroduction).
	If necessary, do you consider the following measures to be justified for protection from wildlife damage in urban areas? (yes/unsure/no)	Feeding bans Containment through structural measures Scaring away by acoustic and visual stimuli Relocation Containment through poisoning Containment through trapping/shooting

**Table A2:** Results from the ANOVA (dependent variable: OS). Deviance residuals, residuals Df, and deviation (Resid Df, Resid. Dev), as well as p-value, are shown for the independent variables. In this respect, interactions between two variables were included in the model; they are marked by colons (:). GE—gender, A—age, R—residency (depending on the size of town), PE—professional engagement, VE—voluntary engagement, HE—hunting engagement, RH—risk of habitat destruction/overlap, RD—risk of damage, RI—risk of infection, RF—risk of fear/prejudice, RE—risk of emotionalization/humanization, RL—risk of lacking knowledge, RNP—risk of unprofessional wildlife care, KS—knowledge Score, EmS—emotion Score, ExS—experience Score).

		Df	Deviance Resid.	Resid. Df	Resid. Dev	*p*	Significance ^1^
	NULL			1272	23,012		
Personal factors							
	GE	3	1663.40	1269	21,349	<0.0001	***
	A	1	2.56	1268	21,346	0.6439	
	R	6	54.92	1215	14,706	0.5969	
	PE	9	1571.50	1259	19,775	<0.0001	***
	VE	2	91.11	1257	19,683	0.0224	*
	HE	4	2002.87	1253	17,681	<0.0001	***
Risk awareness							
	RH	4	91.23	1249	17,589	0.1068	
	RD	4	1184.26	1245	16,405	<0.0001	***
	RI	4	139.18	1241	16,266	0.0206	*
	RF	4	328.20	1237	15,938	0.0183	***
	RE	4	245.31	1233	15,692	0.0004	***
	RL	4	212.95	1229	15,479	0.0014	***
	RNP	4	29.00	1225	15,450	0.6579	
Scores							
	KS	1	399.08	1224	15,051	<0.0001	***
	EmS	1	216.95	1223	14,834	0.0220	***
	ExS	1	68.96	1222	14,766	0.1646	*
	EnS	1	4.26	1221	14,761	0.5505	
Interactions							
	VE:EmS	2	17.95	1213	14,688	0.4721	
	HE:EmS	4	237.78	1209	14,450	0.0006	***

^1^ Significance is indicated as follows: *** <0.001; <0.01; * <0.05; <0.1.

**Table A3:** Results from the ANOVA (dependent variable: EmS). Deviance residuals, residuals Df, and deviation (Resid Df, Resid. Dev), as well as p-value, are shown for the independent variables. In this respect, interactions between two variables were included in the model; they are marked by colons (:). GE—gender, A—age, R—residency (depending on the size of town), PE—professional engagement, VE—voluntary engagement, HE—hunting engagement, KS—Knowledge Score, EnS—Engagement Score, ExS—Experience Score).

		Df	Deviance Resid.	Df, K	Resid. Dev.	*p*	Significance ^1^
	NULL			1286	61,699		
Personal factors							
	GE	3	2951.5	1282	50,081	<0.0001	***
	A	1	10.1	1281	50,071	0.5868	
	PE	9	1394.5	1272	48,676	0.0070	***
	VE	2	104.3	1270	48,572	0.2183	
	HE	4	3340.3	1266	45,231	<0.0001	***
	R	6	633.8	1260	44,598	0.0053	**
Scores							
	KS	1	5.7	1259	44,592	0.6845	
	EnS	1	1538.3	1258	43,054	<0.0001	***
	ExS	1	8666.8	1285	53,032	<0.0001	***

^1^ Significance is indicated as follows: *** <0.001; ** <0.01; <0.05; <0.1.
