# Supplementary material for: Human–Wildlife Interaction—A Social Survey
Source: Animals (Basel). 2024 Mar 5;14(5):808. doi: 10.3390/ani14050808 (PMC10930541; doi:10.3390/ani14050808)
Supplement: Supplementary file 1 [file animals-14-00808-s001.zip › animals-2764218-supplementary.pdf]

animals

Article

Human-Wildlife-Interaction – A SocialSurvey

Lara-Luisa Grundei [1†], Franziska M. Schöttes [2†], Friederike Gethöffer [2,*], Daniel Tost [2], Laurin Kluge [1], Ursula Siebert [2] and Michael Pees [1]

[1] Department of Small Mammal, Reptile and Avian Medicine and Surgery, University of Veterinary Medicine Hanover, Hanover, Germany
[2] Institute for Terrestrial and Aquatic Wildlife Research, University of Veterinary Medicine Hannover, Foundation, Bischofsholer Damm 15, 30173 Hannover, Germany
* Correspondence: friederike.gethoeffer@tiho-hannover.de
† These authors contributed equally to this work.

Supplementary material

Table S1. Output of questionnaire used for this study.

Questionnaire section	Quest. number	Used for score	Question	Question type	n	Answer options	Answers given	
**Personal data**	A2	-	What is your gender?	Single choice	1569	Male	26.7%	
						Female	71.3%	
						Diverse	0.8%	
						N/A (no answer)	1.2%	
	A3	-	What is your year of birth?	Free text	1497	Number field	**Age in years**	**%**
							<20	1.3
							20–39	36.1
							40–59	41.6
							>60	16.4
							N/A	4.6
	A4	-	Where do you live?	Single choice	1569	I live in Germany	97.8%	
						I live outside of Germany	1.8%	
						N/A	0.4%	
	A4a1	-	What is the ZIP code of your town?	Free text, follow-up question, if A4a1 was	1533	Number field	**Residence**	**%**
							<5000 inhabitants	9.7

				answered with "I live in Germany"			5000≤10,000 inhabitants	10.6
							10,000≤20,000 inhabitants	15.8
							20,000≤50,000 inhabitants	21.0
							50,000≤100,000 inhabitants	9.6
							100,000≤500,000 inhabitants	9.8
							>500,000 inhabitants	14.6
							N/A	8.9
	A5	-	Do you work with (wild) animals?	Single choice	1569	Yes	41.3%	
						No	56.5%	
						N/A	2.2%	
	A5a1	-	Which professional background is most likely to apply to you?	Single choice, follow-up question, if A5 was answered with "yes"	648	Veterinary medicine /care	55.7%	
						Nature/species conservation	12.4%	
						Public authority	3.2%	
						Hunting/forestry	10.3%	
						Agriculture	2.8%	
						Natural science research	4.8%	
						Other	9.9%	

						N/A	0.9%
	A5a2	-	Which professional background is most likely to apply to you?	Free text, follow-up question, if A5a1 was answered with „other".	64	Free text	
	A7	-	Do you own a hunting license?	Single choice	1569	Yes, I hunt professionally.	1.9%
						Yes, I hunt privately.	15.7%
						Yes, but I am not active.	5.7%
						No, I don't own a hunting license.	76.0%
						N/A	0.7%
	A6	-	Do you do voluntary work with (wild) animals?	Single choice	1569	Yes	43.1%
						No	54.1%
						N/A	2.8%
**Knowledge (r = right answer, w = wrong answer)**	B2	-	What applies if an animal is subject to hunting laws?	Multiple choice	1569	If an injured animal is found, the hunter in charge must be informed. (r)	89.6%
						If an injured animal is found, the animal may be taken to a veterinary practice without notifying the hunter in charge. (w)	10.7%
						Taking an animal with you is a punishable offence. (r)	51.1%
						N/A	1.1%

	B3	KS	Who is legally responsible for an injured wild animal that is not subject to hunting laws?	Single choice	1569	The person who finds and takes the animal (r)	39.4%	
						Veterinary Office (w)	31.6%	
						Veterinarians (w)	3.4%	
						Fire brigade (w)	3.1%	
						Police (w)	10.5%	
						Animal shelter (w)	4.2%	
						N/A	7.8%	
	B4	KS	When is it legal to take a wild animal from the wild?	Multiple choice	1569	For permanent care when it would no longer cope in nature (w).	29.4%	
						For optimal, more pleasant wintering.(w)	3.6%	
						For health care and reintroduction into the wild. (r)	94.1%	
						If the animal is in danger from predators. (w)	2.1%	
						N/A	2.0%	
	B5	-	Who in Germany mainly pays for the care/treatment of native wild animals? Sort the following terms in descending	Ranking 1-5	1569	Private persons	3.4	average rank
						Veterinarians	2.9	average rank
						Wildlife sanctuaries and associations	4.6	average rank
						State	1.7	average rank
						Municipality	2.5	average rank

			order from high to low financial expenditure:				
	B6	KS	Are veterinarians obliged to treat wild animals free of charge?	Single choice	1569	Yes (w)	5.2%
						In case of emergency (w)	23.0%
						No (r)	68.3%
						N/A	3.5%
	B7	KS	Which of the following diseases belong to zoonoses? (Zoonoses = diseases that can be transmitted between animals and humans)	Multiple choice	1569	African Swine Fever (w)	18.7%
						Rabies (r)	91.5%
						Echinococcosis (fox tapeworm) (r)	86.3%
						Dermatophytosis (skin fungus) (r)	62.5%
						Scabies (Mites) (r)	52.6%
						Aujeszky's Disease (w)	11.6%
						Leptospirosis (r)	38.4%
						Tularemia (rabbit fever) (r)	35.0%
						N/A	3.2%
**Experience**	C2	ExS	What kind of encounters have you had with wildlife so far?	Multiple choice	1569	Observation	97.6%
						Feeding	63.9%
						Touch	65.0%
						Damage	27.3%
						Zoonosis	6.6%
						Attack	6.2%
						Harassment	7.8%

					I have not had an encounter.	0.3%		
					N/A	0.2%		
C2d1	-	Which animal species caused this damage?	Free text, follow-up question if "Damage" was chosen in C2.	416	Free text	Mostly wild boar		
C2e1	-	Which zoonoses were involved?	Free text, follow-up question if "Zoonosis" was chosen in C2.	93	Free text	Mostly ectoparasites		
C2f1	-	By which animal species were you attacked?	Free text, follw-up question, if "Attack" was chosen in C2	94	Free text	Mostly mammals		
C2g1	-	Which animal species were you harassed by?	Free text, follow-up question if "Harrassment" was chosen in C2.	117	Free text	Mostly insects, predatory mammals, birds		
C3	ExS	Have you ever found a wild animal in need of help?	Single choice	1569	Yes	73.0%		
					No	26.6%		
					N/A	0.4%		
C3a1	ExS	You stated that you have found a wild animal in need of help once or several times. Please	Matrix, follow-up question if C3 was answered with „yes".	1146	**Animal group**	**Taken care of by myself (%)**	**Handed over to a specialist (%)**	**Left in nature (%)**
					Canines (e.g., foxes, raccoon dogs)	8.3	15.1	14.1

			indicate how you reacted for all applicable animal groups.			Felines (e.g., lynxes, wildcats)	1.8	9.3	9.1
						Lagomorpha (e.g., brown hares, wild rabbits)	13.1	17.2	12.8
						Rodents (e.g., mice, dormice)	19.5	14.4	14.9
						Insectivores (e.g., hedgehogs)	37.7	28.5	8.4
						Songbirds (e.g., swallows, crows)	32.9	36.5	10.3
						Birds of prey (e.g., falcons, owls)	8.2	31.0	5.0
						Waterfowl (e.g., ducks, geese)	11.9	11.9	8.6
						Bats	7.6	23.3	8.6
						Cloven-hoofed animals (e.g., roe deer, wild boar)	9.1	12.8	10.0
						Reptiles (e.g., lizards, turtles)	5.2	13.5	9.6
						Amphibians (e.g., frogs, toads)	5.9	8.0	18.6
						Other	6.7	9.7	8.6
**Emotion**	C4	EmS	Rate the following animal species according to your feelings towards them:	Matrix: Very negative (1) / rather negative (2) / partly positive, partly negative (3) / rather positive (4) / very positive (5)	1569	Norway rat (*Rattus norvegicus*)	3	median	
						Feral pigeon (*Columba livia* forma *domestica*)	3	median	
						Pine marten (*Martes foina*)	4	median	
						Blue tit (*Cyanistes caeruleus*)	5	median	
						Red squirrel (*Scirurus vulgaris*)	5	median	
						Common crow (*Corvus corone*)	4	median	
						European hedgehog (*Erinaceus europaeus*)	5	median	
						Common buzzard (*Buteo buteo*)	5	median	
						Mallard (*Anas platyrhynchos*)	5	median	

					Slow worm (*Anguis fragilis*)	5	median
	C5	-	Sort the following interactions in descending order according to your personal attitude and the feeling of happiness associated with them.	Ranking 1-9	1569	Watching a wild animal	8.1	average rank
						Feeding a wild animal	4.3	average rank
						Photographing a wild animal	6.3	average rank
						Touching a wild animal	3.0	average rank
						Helping a wild animal in need	6.7	average rank
						Donating to conservation programmes	4.3	average rank
						Actively helping with conservation programmes	5.1	average rank
						Getting politically involved in conservation programmes	3.7	average rank
						Visiting a wildlife park	4.4	average rank
	B12	EmS	To what extent do the following feelings towards some wild animals apply to you? Please rate.	Slider 1-5 (1 = not at all true, 5 = completely true)	1569	Fear of illness/risk of infection	2	median
						Fear of attack	2	median
						Fear of harassment/harm	1	median
						Disgust	1	median
**Opinion**	C8	OS	To what extent do you agree with the following statements?	Matrix: I strongly disagree (1) / I disagree (2) / I partly agree and partly disagree(3) / I agree (4) / I strongly agree (5) / N/A (0)	1569	The care and reintroduction of an injured wild animal makes sense for animal welfare reasons.	5	median
						An injured wild animal that cannot be reintroduced to the wild should be euthanised for welfare reasons.	3	median

					The keeping of native wild animals in wildlife parks is justified for educational purposes.	4	median	
					The keeping of native wild animals in specialist facilities is justified for species conservation purposes (breeding programmes, reintroduction).	4	median	
C10	-	Where do you see the most problems in the interaction between wildlife and humans?	Slider 1-5 (1 = no problems at all, 5 = very big problems)	1569	Habitat overlap	5	median	
					Damage caused by wild animals	3	median	
					Transmission of diseases	2	median	
					Fear/prejudice towards specific wild animals	4	median	
					Emotionalisation/humanization of human-wildlife contact	4	median	
					Lack of knowledge/ lack of education about wildlife	5	median	
					Care or taking in of wild animals by non-professional persons	4	median	
C11	OS	If necessary, do you consider the following measures to be justified to	Matrix (Yes / Unsure / No)	1569	**Measure**	**yes (%)**	**unsure (%)**	**no (%)**
					Feeding bans	68.5	14.9	16.7
					Containment through structural measure	41.8	33.0	25.2

			protect against wildlife damage in urban areas?			Scaring away by acoustic and visual stimuli	41.6	27.0	31.4
						Relocation	48.3	32.7	19.1
						Containment through poisoning	7.0	11.5	81.5
						Containment through trapping/shooting	26.0	20.3	53.8
**Engagement**	B13	EnS	Have you ever been privately involved in native species conservation programs?	Multiple choice	1569	Yes, financially	48.7%		
						Yes, voluntary work / active participation	45.8%		
						Yes, monitoring / observations	39.1%		
						Yes, politically	36.8%		
						No	18.2%		
						N/A	1.8%		
	C13	EnS	How much money would you be willing to pay to a veterinary practice/wildlife center for the care of a wild animal you have found?	Single choice	1569	€0	6.8%		
						€0-10	5.3%		
						€10-50	33.7%		
						€50-100	27.9%		
						€ >100	17.9%		
						N/A	8.4%		
	C12	EnS	To what extent would you be willing to get involved with	Multiple choice	1569	Financially	49.3%		
						Voluntary work	71.6%		
						Politically	33.0%		
						Through further education	60.3%		

			native wildlife in the future?			Own action in private space (e.g., garden design)	88.7%
						None of these measures	1.2%
						N/A	1.8%
**Questionnaire for volunteers**	F1	-	You have indicated that you care for wild animals. What are the three most common groups of animals you care for?	Multiple choice	671	Canines (e.g., foxes, raccoon dogs)	10.9%
						Felines (e.g., lynxes, wildcats)	2.8%
						Lagomorpha (e.g., brown hares, wild rabbits)	17.0%
						Rodents (e.g., mice, dormice)	25.8%
						Insectivores (e.g., hedgehogs)	46.5%
						Songbirds (e.g., swallows, crows)	51.3%
						Birds of prey (e.g., falcons, owls)	20.4%
						Waterfowl (e.g., ducks, geese)	11.9%
						Bats	7.6%
						Cloven-hoofed animals (e.g., roe deer, wild boar)	13.6%
						Reptiles (e.g., lizards, turtles)	3.3%
						Amphibians (e.g., frogs, toads)	6.6%
						N/A	5.7%
	F2	-	How many wild animals do you care for per year on average?	Single choice	666	0-10	44.4%
						10-50	21.3%
						50-100	11.9%
						100-500	8.3%
						>500	4.5%

					N/A	9.6%	
F3	-	How do you finance the care of the animals?	Multiple choice	671	Donations	28.5%	
					Private	82.6%	
					Public support	7.8%	
					N/A	9.6%	
F4	-	How high do you estimate the average financial expenditure per animal to be cared for?	Free text	359	Number field	€0-50	
F5	-	How high do you estimate the average time expenditure per animal and day?	Single choice	664	0-60 minutes	38.4%	
					1-2 hours	25.5%	
					2-5 hours	15.8%	
					5-8 hours	4.0%	
					>8 hours	3.5%	
					N/A	12.8%	
F6	-	To what extent do the following issues affect your volunteer work?	Matrix: This does not affect my voluntary work at all (1) / this does not really affect my voluntary work (2) / this partly affects my voluntary work (3) / this affects	644	Lack of information/lack of knowledge on the part of the finders	5	median
					Lack of financial support from authorities	5	median
					Lack of cooperation from public authorities	4	median
					Inadequate care by veterinarians	3	median
					Poor recognition of the activity	3	median

			my voluntary work (4) / this affects my voluntary work very much (5)		Rising animal numbers	4	median
F7	-	Would you welcome mandatory accredited further training for volunteer wildlife carers?	Single choice	671	Yes	81.7%	
					No	14.2%	
					N/A	4.2%	
F8	-	What do you think should be improved most urgently in the future? Sort the sentences in descending order of importance.	Ranking 1-6	671	Educating the population about native wildlife	4.7	average rank
					Training of veterinarians on native wild animals	3.6	average rank
					Financial support	3.9	average rank
					Cooperation with authorities	2.9	average rank
					Networking between veterinarians and volunteers	3.5	average rank
					Simplification of the legal requirements	2.6	average rank

**Free Text**	D1	-	What questions do you have regarding the problems in dealing with native wildlife that you think should be discussed?	Free text	1569	Free text

Table S2. Variables used in the statistical model. Here, details on the variables shown in tables A2 and A3 are given.

Variable	Definition	Range	Categories
**Gender (GE)**	male, female, diverse	male, female, diverse, N/A	
**Age (A)**	year of birth of participants	1938-2006	-
**Residence (R)**	town residence	-	▪ a (<5000 inhabitants)<br>▪ b (5000 ≤10,000 inhabitants)<br>▪ c (10,000≤20,000 inhabitants)<br>▪ d (20,000≤50,000 inhabitants)<br>▪ e (50,000≤100,000 inhabitants)<br>▪ f (100,000≤500,000 inhabitants)<br>▪ g (≥ 500,000 inhabitants)

**Professional Engagement (PE)**	field of activity	-	▪ A (local authority)<br>▪ B (hunting/forestry authority)<br>▪ C (agriculture)<br>▪ D (nature conservation)<br>▪ E (scientific research)<br>▪ F (veterinary medicine)<br>▪ G (N/A)<br>▪ H (other)
**Voluntary Engagement (VE)**	engaged with animals		yes / no
**Hunting Engagement (HE)**	hunting license holder		▪ A (yes, not active)<br>▪ B (yes, professional)<br>▪ C (yes, voluntarily)<br>▪ D (N/A)<br>▪ E (no)

**Evaluation of risks:**			
**Habitat crossover (RH)**		1-5	
**Human wildlife interaction (RI)**		1-5	
**Disease transmission (RD)**		1-5	
**Fear / prejudice (RF)**		1-5	
**Emotionalization / humanization (RE)**		1-5	
**Lack of knowledge (RL)**		1-5	
**Wildlife care by non-professionals (RNP)**		1-5	
**Emotion Score (EmS)**	Consists of two questions	-28 – 28	-
**Engagement-Score (EnS)**	Consists of three questions	-1 –10	-
**Experience-Score (ExS)**	Consists of three questions	-4 – 17	-
**Knowledge-Score (KS)**	Consists of four questions	0 – 14	-
**Opinion-Score (OS)**	Consists of two questions	-14 – 14	-

Table S3. Grouped wildlife species for wildlife survey dataset

**Geese and ducks**	Greylag goose (*Anser anser*), Canada goose (*Branta canadensis*), Egyptian goose (*Alopochen aegyptiaca*), Eurasian wigeon (*Mareca penelope*), Eurasian teal (*Anas crecca*), mallard (*Anas platyrhynchos*)
**Cloven-hoofed game**	European fallow deer (*Dama dama*), European mouflon (*Ovis gmelini musimon*), roe deer (*Capreolus capreolus*), red deer (*Cervus elaphus*), wild boar (*Sus scrofa*)
**Neozoa**	Coypu (*Myocastor coypus*), raccoon (*Procyon lotor*), raccoon dog (*Nyctereutes procyonoides*), American mink (*Neogale vison*), Canada goose (*Branta canadensis*), Egyptian goose (*Alopochen aegyptiaca*)

**Birds**	Eurasian magpie (*Pica pica*), Common raven (*Corvus corax*), Carrion crow (*Corvus corone*), pheasant (*Phasianus colchicus*), Grey partridge (*Perdix perdix*), Greylag goose (*Anser anser*), Canada goose (*Branta canadensis*), Egyptian goose (*Alopochen aegyptiaca*), Eurasian wigeon (*Mareca penelope*), Eurasian teal (*Anas crecca*), mallard (*Anas platyrhynchos*), Eurasian collared dove (*Streptopelia decaocto*), wood pigeon (*Columba palumbus*)
**Mammals**	European hare (*Lepus europaeus*), European rabbit (*Oryctolagus cuniculus*), red fox (*Vulpes vulpes*), European badger (*Meles meles*), coypu (*Myocastor coypus*), Eurasian beaver (*Castor fiber*), raccoon (*Procyon lotor*), raccoon dog (*Nyctereutes procyonoides*), American mink (*Neogale vison*), European wildcat (*Felis silvestris*), wolf (*Canis lupus*), Eurasian lynx (*Lynx lynx*), European fallow deer (*Dama dama*), European mouflon (*Ovis gmelini musimon*), roe deer (*Capreolus capreolus*), red deer (*Cervus elaphus*), wild boar (*Sus scrofa*)
**Predatory mammals**	Red fox (*Vulpes vulpes*), European badger (*Meles meles*), racoon dog (*Nyctereutes procyonoides*), Raccoon (*Procyon lotor*), mink (*Neogale vison*), European wildcat (*Felis silvestris*), wolf (*Canis lupus*), Eurasian lynx (*Lynx lynx*)
**Species under special protection**	Eurasian beaver (*Castor fiber*), European wildcat (*Felis silvestris*), wolf (*Canis lupus*), Eurasian lynx (*Lynx lynx*)
**Management species (city)**	Carrion crow (*Corvus corone*), European rabbit (*Oryctolagus cuniculus*), red fox *(Vulpes vulpes)*, coypu (*Myocastor coypus*), raccoon (*Procyon lotor*), wild boar (*Sus scrofa*)
